# Cerebral Venous Sinus Thrombosis with Bilateral Abducens Palsy in a Patient with Heterozygous Prothrombin G20210A Mutation

**DOI:** 10.7759/cureus.6124

**Published:** 2019-11-11

**Authors:** Soujanya Sodavarapu, Saiyed W Ali, Megha Goyal

**Affiliations:** 1 Internal Medicine, San Joaquin General Hospital, French Camp, USA

**Keywords:** cerebral sinus venous thrombosis (csvt), prothrombin gene mutation, prothrombin g20210a gene, headache, cerebral sinus thrombosis, anti-coagulation therapy, diplopia, bilateral abducens nerve palsy

## Abstract

We present a rare case of cerebral venous thrombosis in a patient with heterozygous mutation of the prothrombin G20210A gene. A 24-year-old Caucasian male complained of sudden onset diplopia due to bilateral abducens palsy and throbbing headache with pulsatile tinnitus. Computed tomography revealed extensive thrombus in the right sigmoid sinus, right transverse sinus, and the superior sagittal sinus. Except for the prothrombin mutation, no other predisposing risk factors were found. The patient was treated with heparin, later transitioned to enoxaparin and warfarin, and subsequently, the symptoms improved on follow-up. This case reveals the rare occurrence of cerebral venous thrombosis in a patient having prothrombin gene G20210A mutation alone. Early diagnosis and treatment can lead to a good prognosis.

## Introduction

Cerebral venous sinus thrombosis (CVST) is a cerebrovascular disease that can occur in patients with predisposing risk factors for deep vein thrombosis. Less commonly, it can be seen in patients with inherited conditions such as deficiency of protein C, protein S, antithrombin III, and factor V Leiden mutation [1,2]. Mutation in the prothrombin gene resulting in a heterozygous state is associated with increased levels of prothrombin [2]. Cerebral venous thrombosis can occur in patients who have other predisposing risk factors for deep vein thrombosis in addition to such inherited mutations [2]. Here, we are reporting a case of heterozygous prothrombin gene mutation in a young male without other predisposing risk factors who had been diagnosed with extensive cerebral venous thrombosis and abducens palsy.

## Case presentation

A 24-year-old Caucasian male with no significant past medical history presented to the emergency room with a complaint of sudden onset double vision and headache with worsening intensity associated with non-bloody emesis for four days. He described his double vision as seeing two images of a single object and unable to move his eyes outward bilaterally. He related his headache as throbbing in character, severe in intensity, and diffusely involving his entire skull with radiation down his neck. He also noted worsening of his headache with cough and Valsalva, associated with pulsatile ringing in the ears.

**Figure 1:**
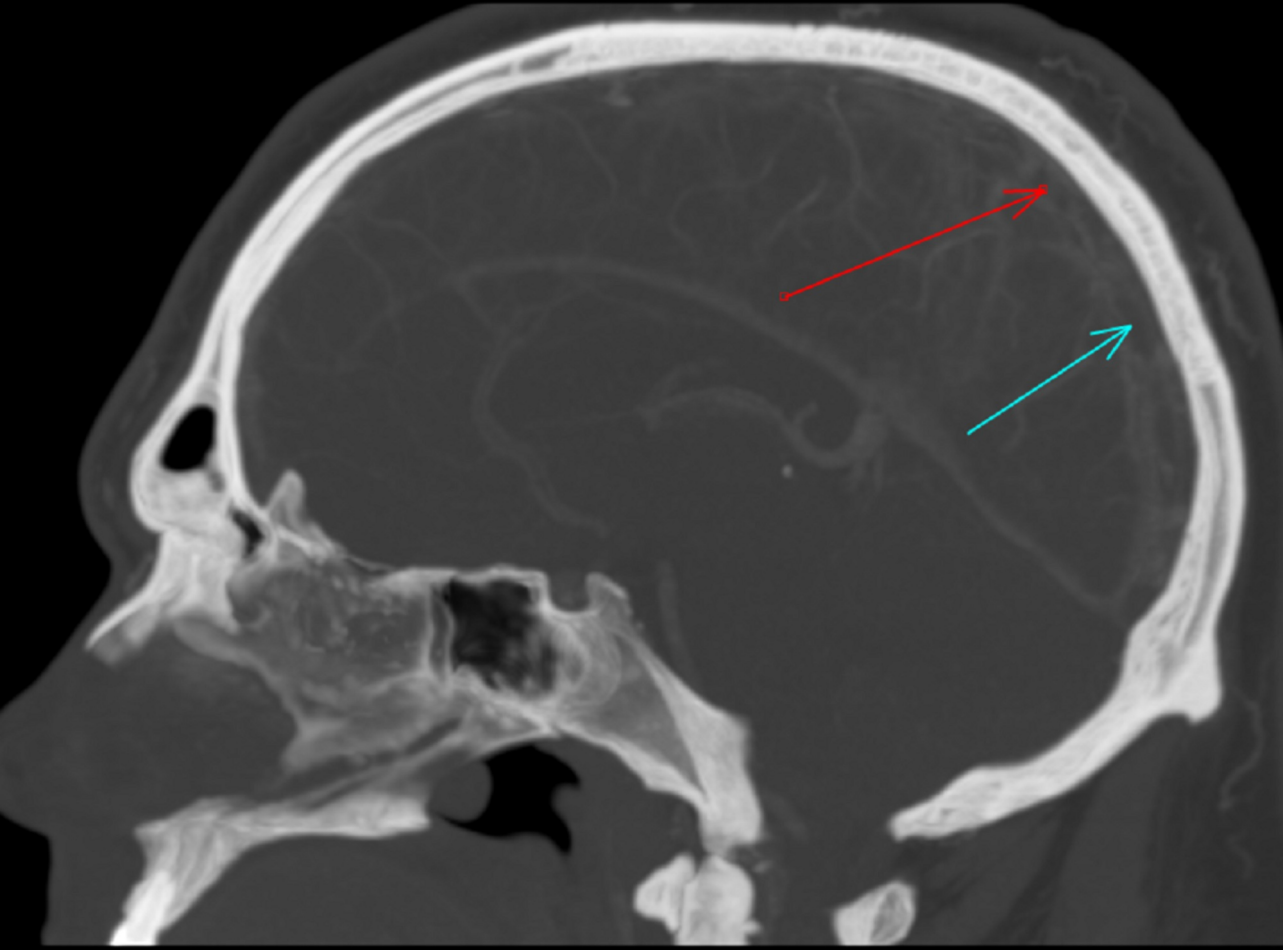
Pre therapy computed tomography with contrast, arrows showing superior venous thrombosis

**Figure 2:**
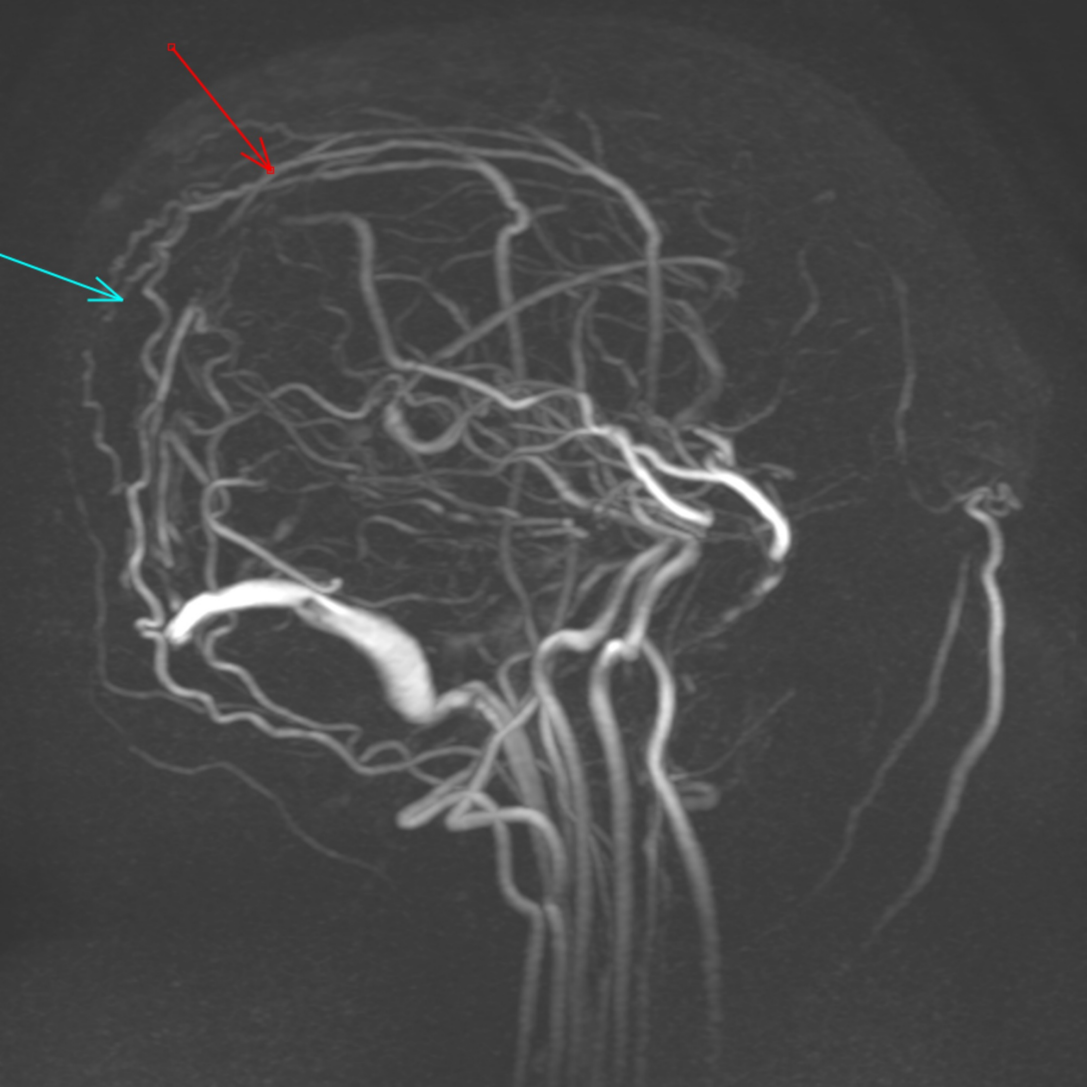
Magnetic resonance venography of brain, arrows showing absence of the superior sagittal sinus due to thrombosis

Vital signs were within normal limits. Neurological examination revealed bilateral abducens nerve palsy. He underwent a dilated fundoscopic examination which did not show any evidence of papilledema. Computed tomography (CT) of the head was done, which showed an extensive thrombus from the proximal portion of the right internal jugular vein extending into the right sigmoid sinus, right transverse sinus, following into the confluence of sinuses and extending into the superior sagittal sinus (Figure 1). Magnetic resonance venography (MRV) showed the absence of blood flow in the superior sagittal sinus (Figure 2). He underwent a CT of chest/abdomen/pelvis, which did not show any evidence of malignancy. There were no signs of sinusitis, sepsis, head injury, or mechanical precipitants. He has shown no indication of systemic or intracranial infection during this admission; therefore, a lumbar puncture was not done.

**Figure 3:**
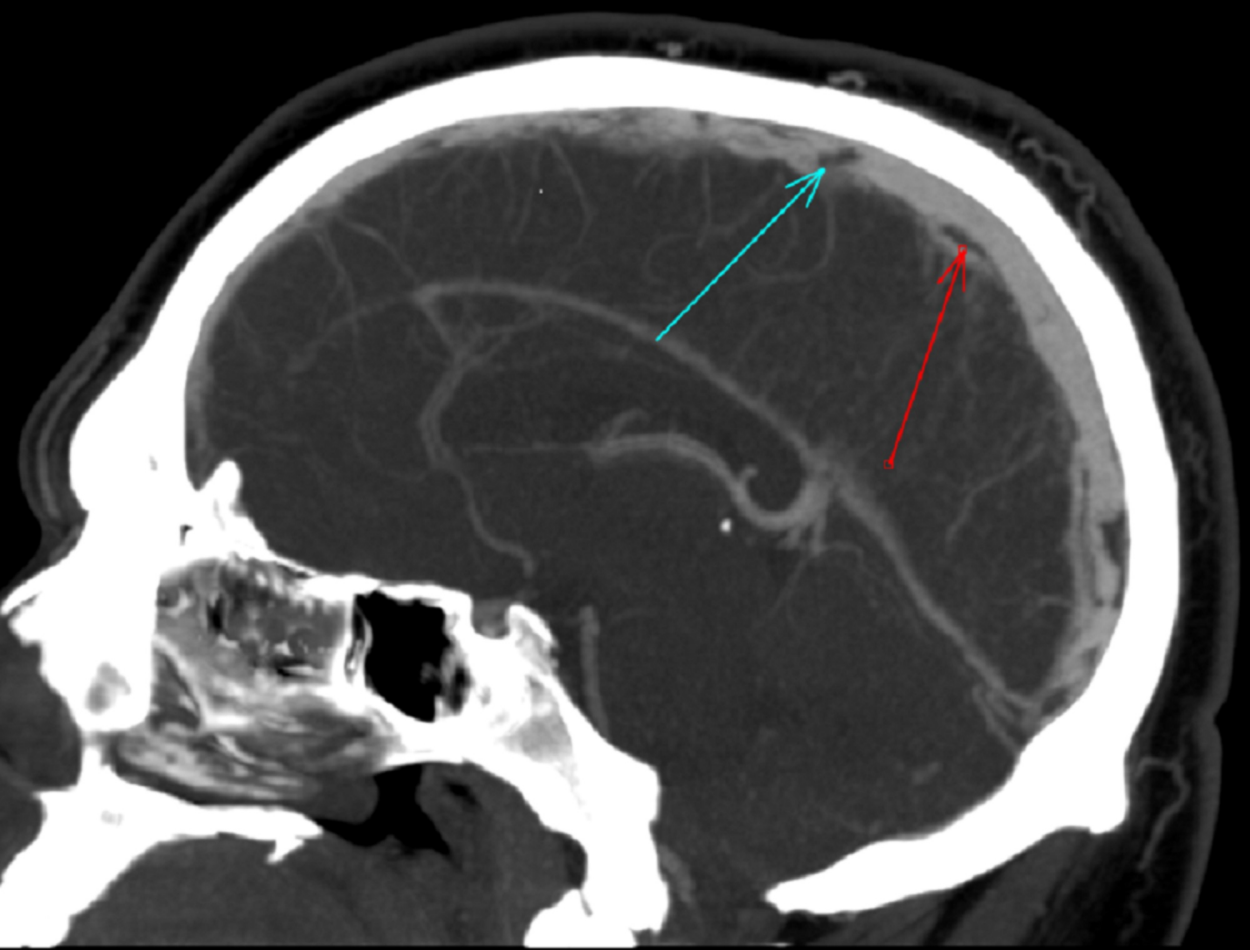
Post therapy, sagittal view of computed tomography of the brain with contrast, arrows showing dissolving clots and re-canalization in the superior sagittal sinus

He was admitted to the hospital for control of his headache and nausea, which improved with intravenous (IV) analgesics and antiemetics. He was started on IV heparin, transitioned to warfarin and enoxaparin during his hospital course, and was subsequently discharged home on warfarin and enoxaparin. He had extensive hypercoagulable workup, which was pending at the time of discharge. The results for factor C, factor S, factor V Leiden, homocysteinemia, and antiphospholipid antibody were negative except for heterozygous mutation of the prothrombin G20210A gene. D-dimer was not checked in our patient, but when elevated, it is supportive of the diagnosis. However, normal levels do not rule out the diagnosis. This is in contrast to other causes of venous thromboembolism where d-dimer tends to have a very high negative predictive value. The patient had a repeat CT of the head with contrast in seven days, which showed dissolving cerebral venous thrombosis (Figure 3). His diplopia and headache improved at the time of a repeat CT scan. He was able to move eyes in all directions, still had a constant headache for which was started on topiramate. On a month follow-up, his vision continued to improve and his headache resolved. 

## Discussion

Cerebral venous sinus thrombosis (CVST) is a rare and uncommon cerebrovascular disease that affects 3 to 4 in a million people in a year [1]. There is a significant overlap of the many risk factors for CVST and those for venous thromboembolism (VTE): cancer, obesity, genetic thrombophilia, trauma, infection, and prior neurosurgery [2]. Predisposing factors have been recognized in up to 80% of patients who develop CVST [3]. Hereditary thrombophilia's as a cause of cerebral venous thrombosis may be seen in 10-15% of patients [4]. Bilateral abducens palsy is rare in CVST, and on literature review has not found any case reports of it in CVST due to prothrombin gene mutation. A case has been described by Buljan et al. in puerperium patients with protein S deficiency [5]. Another example was described as a complication of chronic suppurative otitis and mastoiditis. Other causes have been from subarachnoid hemorrhage, Diane syndrome, idiopathic intracranial hypertension, and diabetes [5]. 

We present a rare cause of cerebral venous thrombosis with abducens palsy in a male patient with heterozygous mutation of the prothrombin G20210A gene. A single substitution of guanine for adenine at nucleotide 20210 on the prothrombin gene located on chromosome 11p generates increased levels of prothrombin [2,3]. This mutation causes elevated levels of prothrombin the precursor to thrombin, which is an essential component of the common pathway in coagulability [6,7]. In many cases of prothrombin G20210A heterozygous deficiency, cerebral venous thrombosis occurred in the setting of other prothrombic states such as pregnancy or with the use of oral contraceptives [2,6]. In a meta-analysis done by Gonzalez et al., who reviewed a total of 868 cases from 19 studies of cerebral venous thrombosis and 3,981 controls, 103 of the 868 patients with cerebral venous thrombosis had prothrombin G20210A [8]. In most of the studies, the affected persons were mostly females with either pregnancy or oral contraceptives and were rare in males [8]. Our case is unique as the patient is a male and did not have any other predisposing factors such as infection, trauma, cancer, or prior neurosurgery and at presentation had bilateral abducens palsy. 

The clinical signs and symptoms in CVST include headache, seizures, focal neurologic deficits, and neuro-ophthalmologic features such as papilledema, diplopia, loss of vision, and constriction of the visual field [9]. All of these occur due to elevated intracranial pressure and eventual ischemia or intracranial hemorrhage. Headache is generally the first and most frequent symptom seen in cerebral venous thrombosis and is seen in nearly 90% of the patients [10]. In some patients, the headache may be of thunderclap type, which resembles that due to subarachnoid hemorrhage [11]. The signs and symptoms that are seen in patients with cerebral venous thrombosis can be grouped into four patterns [12]. Cavernous sinus thrombosis causes eye pain, bulging of eyes, and paralysis of extraocular muscles. Patients with intracranial hypertension alone have complaints of headaches associated with nausea and vomiting, papilledema, tinnitus, and decreased vision. Patients with superficial venous thrombosis and lesions in parenchyma usually have focal neurological deficits along with seizures. Patients with deep venous thrombosis having edema of basal ganglia and thalamus can present with gaze disorder, encephalopathy, or even coma in severe cases [12]. The sixth cranial nerve, due to its extended intracranial course, is susceptible to increased intracranial pressure from CVST [6]. Patients who have inferior petrosal sinus thrombosis may also have isolated ipsilateral abducens palsy. This complication is observed due to compression of the nerve during its course in Dorello's canal [13]. Compression at a different site is thought to cause bilateral abducens palsy. In the case of subarachnoid hemorrhage, it is believed to be due to both compression and stretching of the bilateral abducens nerves by a thick clot in the prepontine cistern that causes nerve palsy [14]. In our case, the patient chiefly complained of diplopia due to bilateral abducens nerve palsy and severe headache associated with nausea, vomiting, and tinnitus.

Neuroimaging plays a crucial role in the diagnosis of CVST. In the last few decades, there has been an increased number of diagnoses of CVST due to the improvement in neuroimaging studies (CT, magnetic resonance imaging [MRI], CT venography, and MRV), allowing an appropriate treatment with anticoagulant therapy before adverse events occur [9,15]. Good outcomes have been observed in more than 80% of patients [10,16]. The sign seen in approximately 20% of patients is commonly called a triangle sign or cord sign [14,15]. This sign is caused by a recently thrombosed dural sinus or cortical vein, which is identified in a non-contrast CT as a hyperattenuating lesion [16,17]. Another commonly identified sign in patients with cerebral venous thrombosis is the empty delta sign. This sign refers to a thrombus located in the superior sagittal and transverse sinuses seen as a triangular defect in post-contrast imaging [17]. This sign is more common than the previously mentioned cord sign and is seen in 25%-75% of cases of cerebral venous thrombosis [17]. 

Both low-molecular-weight heparin (LMWH) and unfractionated heparin (UFH) can be used to treat CVST. Still, LMWH is more appropriate, except when the patient may need surgical intervention, in which case, anticoagulation should be immediately reversed [9,15,18,19]. A randomized controlled trial was done in 2012 compared to the efficacy and safety of LMWH and UFH in CVST. The authors found that hospital mortality was significantly lower in patients treated with LMWH than in patients treated with UFH [9,15]. A good prognosis is seen in most patients after anticoagulation, but in some patients with severe CVST, who exhibit further deterioration of the disease, intravascular treatment is considered [9]. Recent studies have shown some positive results with the use of direct oral anticoagulants. They would be preferred due to their ease of administration and monitoring, but the data are limited, and more studies need to be conducted to provide definitive guidance to the clinicians [20].

Our patient was discharged on subcutaneous enoxaparin (LMWH) and oral warfarin. LMWH was given until the therapeutic level of the international normalized ratio between 2 and 3 was achieved. In two weeks of follow-up, the patient's abducens palsy resolved and was able to move eyes in all directions; although some diplopia was still present, the headache did improve. At four weeks of follow up, the headache had resolved.

## Conclusions

CVST is a rare disease. It can have many risk factors, including coagulation abnormalities. Prothrombin gene mutation G20210A should be considered in patients diagnosed with CVST, including other coagulation disorders. Abducens palsy could be one of the signs which need to be looked for as well. Treatment with anticoagulation should be immediately started to prevent infarction of the brain and adverse events.
